# Making sense of the virome in light of evolution and ecology

**DOI:** 10.1098/rspb.2025.0389

**Published:** 2025-04-02

**Authors:** Megan A. Wallace, Michelle Wille, Jemma Geoghegan, Ryan M. Imrie, Edward C. Holmes, Xavier A. Harrison, Ben Longdon

**Affiliations:** ^1^ Centre for Ecology and Conservation, Faculty of Environment, Science and Economy, University of Exeter, Penryn, Cornwall, UK; ^2^ Centre for Pathogen Genomics, Department of Microbiology and Immunology, Peter Doherty Institute for Infection and Immunity, University of Melbourne, Melbourne, Victoria, Australia; ^3^ Department of Microbiology and Immunology, University of Otago, Dunedin, New Zealand; ^4^ School of Medical Sciences, University of Sydney, Sydney, Australia

**Keywords:** virome, viruses, virus metagenomics, virus ecology, virus evolution, virus communities

## Abstract

Understanding the patterns and drivers of viral prevalence and abundance is of key importance for understanding pathogen emergence. Over the last decade, metagenomic sequencing has exponentially expanded our knowledge of the diversity and evolution of viruses associated with all domains of life. However, as most of these ‘virome’ studies are primarily descriptive, our understanding of the predictors of virus prevalence, abundance and diversity, and their variation in space and time, remains limited. For example, we do not yet understand the relative importance of ecological predictors (e.g. seasonality and habitat) versus evolutionary predictors (e.g. host and virus phylogenies) in driving virus prevalence and diversity. Few studies are set up to reveal the factors that predict the virome composition of individual hosts, populations or species. In addition, most studies of virus ecology represent a snapshot of single species viromes at a single point in time and space. Fortunately, recent studies have begun to use metagenomic data to directly test hypotheses about the evolutionary and ecological factors which drive virus prevalence, sharing and diversity. By synthesizing evidence across studies, we present some over-arching ecological and evolutionary patterns in virome composition, and illustrate the need for additional work to quantify the drivers of virus prevalence and diversity.

## 1. Introduction

Viruses are ubiquitous across life on earth, but we have much to learn about what determines communities of viruses (i.e. the ‘virome’ or ‘virosphere’) across hosts and ecosystems. Virus community composition can be characterized in different ways: prevalence (proportion of hosts infected), abundance (the viral load of a host/population) and distribution (temporally or spatially) of viruses within those communities. Large-scale metagenomic sequencing projects have expanded our knowledge of the diversity and composition of eukaryotic viromes [1], with the number of published viral metagenomics papers increasing more than sixfold in the last decade, and the number of classified virus species increasing more than threefold (5542 released virus RefSeq genomes on NCBI in September 2014 versus 18 668 in September 2024). This number is expected to increase dramatically following the implementation of discovery models that utilize protein structure as well as sequence data, with a single recent study using an AI-based approach identifying ore than 160 000 novel virus species [2]. Similarly, structural prediction models have the potential to improve our understanding of virus evolution over long timescales as well as host–virus interactions [3,4]. Consequently, the rate of virus discovery is greatly out-pacing virus classification. Despite this revolution in virus discovery, the field is only just beginning to move from being purely descriptive ‘molecular natural history’ to being hypothesis driven.

Over the last decade the metagenomic sequencing of animal, plant and soil-associated bacterial communities—often referred to as microbiome research—has transitioned from a descriptive state towards directed hypothesis testing (see reviews [5,6]). Continuous monitoring of wild populations has allowed the analysis of long-term datasets to study the determinants and fine-scale variation in microbiomes. Examples include global variation in amphibian skin bacterial communities linked to climate [7], variation in the bacterial microbiomes of birds linked to foraging behaviour [8] and seasonality in gut parasite communities [9] and bacterial microbiotas [10] in mammals. In contrast, most virus-focused metagenomic studies can only be interpreted as a single snapshot of the virome of an individual, population, species or environment at a particular point in time and space [5]. Testing explicit ecological and evolutionary hypotheses on the causes and consequences of variation in the virome requires that we (i) integrate extensive spatial and longitudinal virome sampling alongside ecological data, and (ii) embed the virosphere in a whole community context by considering viruses not only as potential zoonotic diseases, but as participants in their wider ecosystems. Collectively, this will allow us to determine their importance in maintaining whole ecosystem functionality and stability [11].

Addressing this knowledge gap is currently hampered by biases in the metagenomic literature, which could lead us to overstate broad-scale patterns or drivers of virome diversity [12]. For example, large databases of host–virus associations [13,14] are biased towards mammalian viruses, and groups such as bats with high research interest. Such biases can lead to dogmas in the literature, for example, it has been suggested that more zoonotic diseases originate from particular host groups because of their inherent immunology or ecology, although in some instances this could simply reflect inherent sampling biases [14]. As a result, compilation of databases is urgently needed for less well sampled groups, as currently being attempted in insects [15].

Understanding the determinants of and barriers to successful cross-species transmission of viruses is crucial to understanding the potential of a virus to emerge in a new species. Identifying the factors that enable or inhibit virus transmission among hosts involves taking a whole ecosystem (i.e. 'one health') approach [16] and has a broad implications for public and agricultural health. For example, the evolutionary and ecological factors that structure species viromes directly influence disease emergence in wildlife [17–20], pollinators [21,22] and livestock [23,24], and have clear connections to spillover into humans. In addition, viruses interact, both directly and indirectly, within ecosystems (e.g. host disease caused by one virus may prevent the transmission of other viruses) [25]. Here, we summarize our current knowledge of the ecological and evolutionary factors determining virome composition, and propose how we can expand this with future research (box 1).

Box 1. **What data do we need for informative studies in virus ecology and evolution?**

To design experiments and sampling schemes that allow the quantification of the ecological and evolutionary factors that structure species’ viromes we need the following.

***Sampling design***

*How can we create balanced sampling from both a virus and host perspective?*

— Viruses:
  - **Identification of the host that a virus is actually infecting**, because of the existence of multiple hosts in metagenomic samples (e.g. the bacterial microbiome, host dietary components and eukaryotic parasites or symbionts). Can be done by comparing novel virus genomes to existing viral phylogenies. Non-host-associated viruses can then be used as an internal control, as they should not be affected by trends in host-associated viruses.
  - **Increased attention to DNA viruses** to ascertain whether there is a dearth of DNA viruses in some ecosystems or groups.
  - **Aim to characterize the within-host diversity of viral communities,** and therefore its drivers, possibly by combining short and long read sequencing [26] to distinguish between co-circulating haplotypes and structural variants.
— Hosts:
  - **Utilizing carefully designed, systematic sampling of species/ecosystems**—using power analyses (with simulations based on existing/pilot data) to determine the number of individuals and species sampled, rather than haphazard approach.
  - **Sampling multiple individuals of a host species and in a variety of habitat types/seasons** to estimate virus prevalence, climatic, seasonal or habitat effects and scale dependencies [27].
  - **Sampling complete food webs/trophic networks/ecosystems**—by considering which systems allow more complete sampling of potential host taxa (e.g. islands [28,29], tree fogging and ponds).
  - **Gathering data from traditionally under-sampled ecosystems**, enabling us to examine the effect of different ecologies and life-history traits on the structure of the virome. Current sampling biases have likely skewed our view of the ecology of even well sampled host virospheres. Predictions of viral sharing [13] or potential host-shifting will not be able to be expanded out of well sampled (e.g. mammalian) groups without more detail on the host range and ecological context of non-mammalian viromes.

***Utilizing species distribution and demographic history data***

*How can we expand the possible virome predictors we can test using public data?*

— Testing drivers of prevalence and diversity by **making use of historic climatic data and data on anthropogenic environmental changes** such as land use change (introduction of agriculture, urbanization) and habitat disturbance levels.
— By **making use of data from public citizen science projects** [30,31], and the expertise of local forums or naturalist communities (e.g. Dipterists Forum, https://dipterists.org.uk; eBird, https://ebird.org), we can examine how more factors (e.g. migration/range shifts, impact virus prevalence and diversity).
— By incorporating public data on the **presence/absence of symbionts or coinfecting macro-parasites** (e.g. Varroa mites with DWV [32]), we can assess their impact on viral prevalence and virome composition—ultimately aiming for data from whole macro, symbiont, microbiome and virome datasets.

***Analysis***

*How can we quantify the effect size of both ecological and evolutionary drivers of species viromes?*

— **By utilizing a mixed-model approach** [33]**, including co-phylogenetic mixed models**, it is possible to draw out both evolutionary and ecological predictors of virus prevalence and host range [34,35].
— By **estimating diversity directly from linear models,** we may be able to quantify the effect of factors on virus diversity, as well as prevalence [36].
— By **accounting for spatial and temporal autocorrelation** in analyses of the drivers of virus prevalence, we not only make our identified drivers more robust but also quantify the influence of spatial and temporal effects.
— **Developing the equivalent tools for the analysis of virome data that are already available for the analysis of bacterial microbiomes.**

## 2. Evolutionary factors driving the composition of species viromes

Viruses, like bacteria and fungi [37], are often preferentially shared between closely related hosts [38], and traits shared between phylogenetically closely related species will shape the composition of the virome. These traits, such as host receptors, physiology and immunity, present a similar environment for a virus and are the result of the history of selection on hosts (and viruses), in part caused by their exposure history [39]. However, the importance of host relatedness does not necessarily present as a linear relationship between susceptibility and host phylogenetic distance. Closely related hosts may have similar levels of susceptibility to a given virus (or group of viruses), independent of their distance from the viruses ‘natural’ host. This ‘clade effect’ can result in viruses being clustered in a patchwork of clades on the host phylogeny [40–42].

This concept also holds true for surveys of viral presence/absence in natural populations. Host phylogenetic relatedness is a significant predictor of the likelihood of viral sharing between primates [43]. As a particular case in point, rabies virus sequences sampled from single viruses across multiple bat host species reveal that cross-species transmission events and successful host shifts are more likely in closely related host species [17,44]. Importantly, in this system, range overlap is less important than phylogenetic relatedness in predicting sustained host shifts compared to spillover events (although current estimates of geographic range are used to test this, rather than historical range). Additionally, host phylogenetic effects have been demonstrated for particular viruses in a range of hosts both experimentally [45–48] and in nature [42], although we do not know how such effects impact virome structure.

Large databases of host–virus associations have also shown an increased proportion of zoonotic viruses in species that are closely related to humans [49], and that species-rich host taxonomic groups harbour more viruses [14]. This again supports the idea that viruses can preferentially jump between closely related host species. In addition, these databases demonstrate that some virus lineages have a greater propensity to change hosts [50] and that viruses with broad host ranges have a greater propensity to jump host [51]. However, an important caveat is that these analyses are based on our current incomplete understanding of global viral diversity [12]. There is also some evidence that host species may vary in their overall susceptibility to viral infection or cross-species transmission [18,45]. However, at least among mammalian viruses, there is no evidence that particular host taxonomic groups are inherently more likely to be virus reservoirs because of host traits. On the contrary, host taxonomic orders with greater species richness simply appear to harbour more diverse viromes, and are therefore more often the source of cross-species transmission events [14,52].

**Figure 1.**
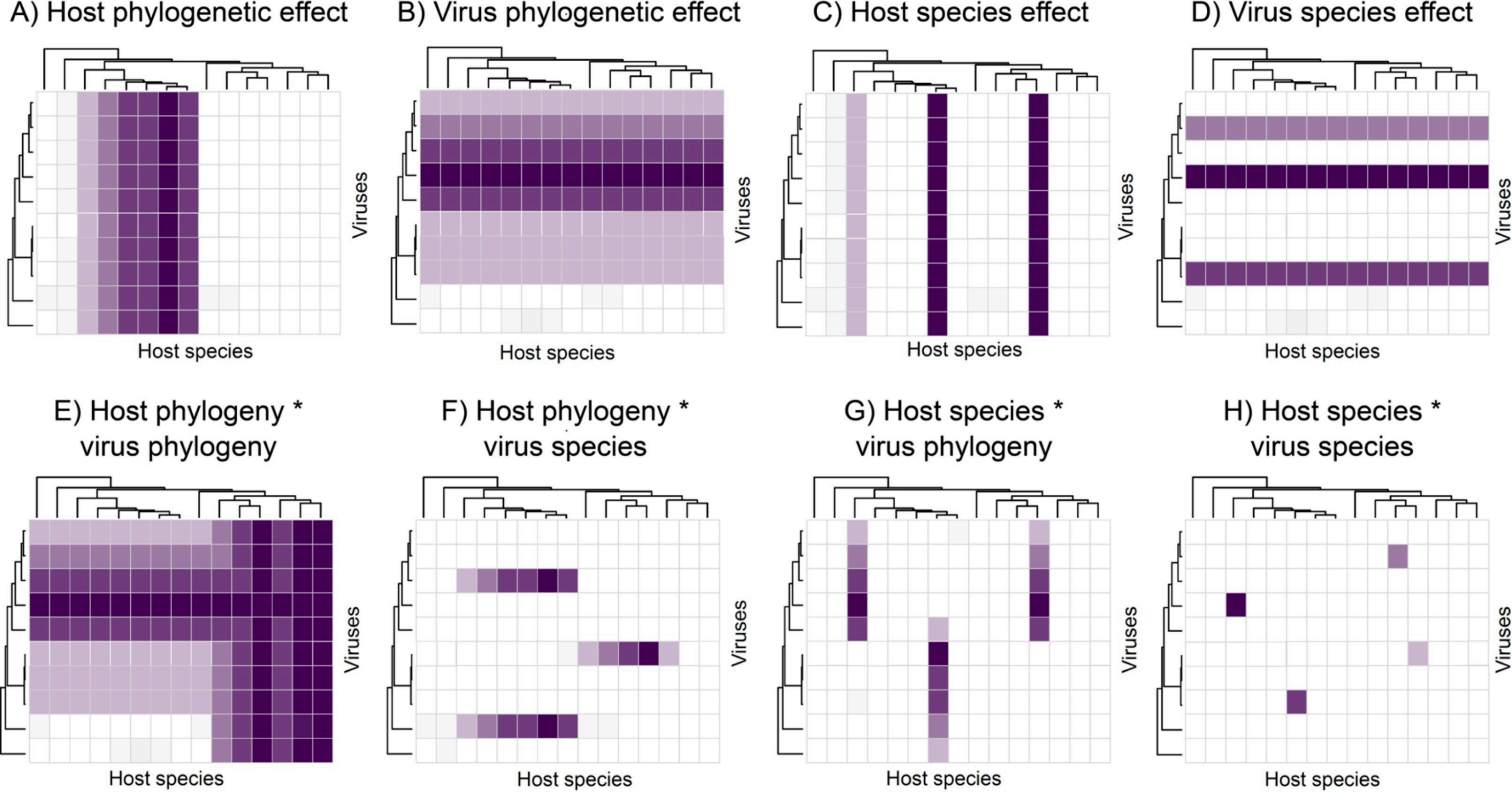
Host and virus species level and phylogenetic effects on virus prevalence and viral host range. The *y*-axis represents a hypothetical virus phylogeny and the *x*-axis a hypothetical host phylogeny. Asterisk indicates model interaction terms. Each panel represents different possible scenarios. (A) The incidence and prevalence of viruses across host species is predictable by host phylogeny (i.e. closely related host species have a similar incidence of viruses). (B) The incidence and prevalence of viruses across host species is predictable by virus phylogeny (i.e. closely related viruses have a similar infectivity across host species). (C) Certain host species are inherently more or less susceptible to viruses, in a way not predictable by the host phylogeny (i.e. due to ecological or physiological traits). (D) Certain viruses are particularly infectious, or not, irrespective of host species, in a way not predictable by virus phylogeny. (E) Related hosts have similar incidences of clades of related viruses (i.e. virus incidence and prevalence is predictable by both host and virus phylogenies). (F) Related hosts have similar incidences of some viruses, but not all, and not in a phylogenetically predictable manner. (G) Related viruses show similar infectivity to only some host species—not all—and not in a way predicted by host phylogeny. (H) Host susceptibility depends on specific host × virus interactions not predictable by either host or virus phylogeny. Based on [34,53].

To quantify the relative importance of host and virus relatedness requires analysis of many related hosts and viruses. The evolutionary drivers of virome composition can be broken down into a series of ‘species level’ and ‘phylogenetic’ effects (figure 1) [34,53]. Host species effects and phylogenetic effects capture how hosts vary in their overall prevalence of viral infection and whether related hosts tend to have similar overall viral prevalence for the host (figure 1A,C). Virus species effects and phylogenetic effects capture how viruses vary in the overall size of their host range, and whether related viruses have similar host ranges (figure 1B,D). By examining these effects it is possible to ask whether some hosts are more susceptible than others, whether some viruses are more generalist than others, and if these traits are similar among related hosts or viruses. In addition, hosts may vary systematically in the composition of their virome, and viruses may vary systematically in the composition of their host range. For example, it is well established that viruses generally transmit more easily between more closely related host species [46] and that host–virus co-divergence also occurs [54], although less commonly than cross-species transmission in many groups [55]. Importantly, both of these processes mean that related hosts (or viruses) will have more similar viromes (or host ranges) [56,57], sometimes referred to as ‘phylosymbiosis’ [58]. Moreover, we expect related hosts (or related viruses) to be more similar in their virome composition (or host range). We can examine these questions by looking at interactions between the terms described above (figure 1) [34].

Interactions between host and virus species-level effects correspond to unique species-by-species interactions in susceptibility or resistance that are not predictable from the relatives of either the host or virus (figure 1H). An interaction between host phylogeny and virus species corresponds to an individual virus being a specialist on (or limited to) specific clades of the host (figure 1F); and an interaction between virus phylogeny and host species corresponds to specific clades of viruses showing similar infectivity on a specific host (figure 1G). The phylogenetic interaction term corresponds to particular clades of the host being more prone to infection by particular clades of the virus—as predicted by co-divergence or near-neighbour preferential host-switching models (figure 1E) [59].

Recent metagenomic sequencing studies are beginning to generate data that can address these questions confirming, for example, that the host phylogeny predicts a significant proportion of variance in the structure of virus communities. As a case in point, the viromes of marine fish are predominantly shaped by the phylogenetic history of their hosts [60], influencing both alpha and beta virome diversity [61]. Likewise, host taxonomy in birds is important in explaining differences in virus community structure [62]. Additionally, host order explains significant variation in the viral richness and prevalence in wild bats, rodents and shrews [18]. Likewise, viral richness in species sampled across an entire island ecosystem clusters by host taxonomy, with viral order explaining the most variation in virus community composition [28]. These studies also demonstrate how we can simultaneously quantify the relative importance of both phylogeny and ecology in determining virome composition and diversity (see also [63]).

Importantly, these studies fit taxonomic groups as categorical/random effects in models, rather than the effect of the host phylogeny directly. A more sophisticated—although data intensive—approach is to simultaneously fit species level and phylogenetic (relatedness) effects (box 1). For example, in a study of 13 bumblebee species and 20 viruses approximately a quarter of the variation in virus prevalence was explained by the evolutionary histories of the hosts and viruses (i.e. the sum of the host and virus phylogenetic effects illustrated in figure 1A,B,E–G) [53]. However, individually each of the host and virus effects explained only a small proportion of the variance in prevalence with large amounts of uncertainty around these estimates, which may reflect a lack of power to detect such effects on a relatively small number of hosts and viruses. Indeed, even when sampling a larger number of hosts and viruses, the best-fit models of the predictors of viral richness and prevalence in wild rodents, bats and shrews explained less than 40% of deviance, highlighting the challenges in accurately explaining the patterns of viral diversity and abundance across species [18]. In addition to aspects of virus ecology and evolution, the analysis of individual sequencing libraries offers the potential to make inferences on aspects of virus population genetics, such as examining the effects of changes in population size on virome composition.

## 3. Ecological drivers of virome composition

**Figure 2.**
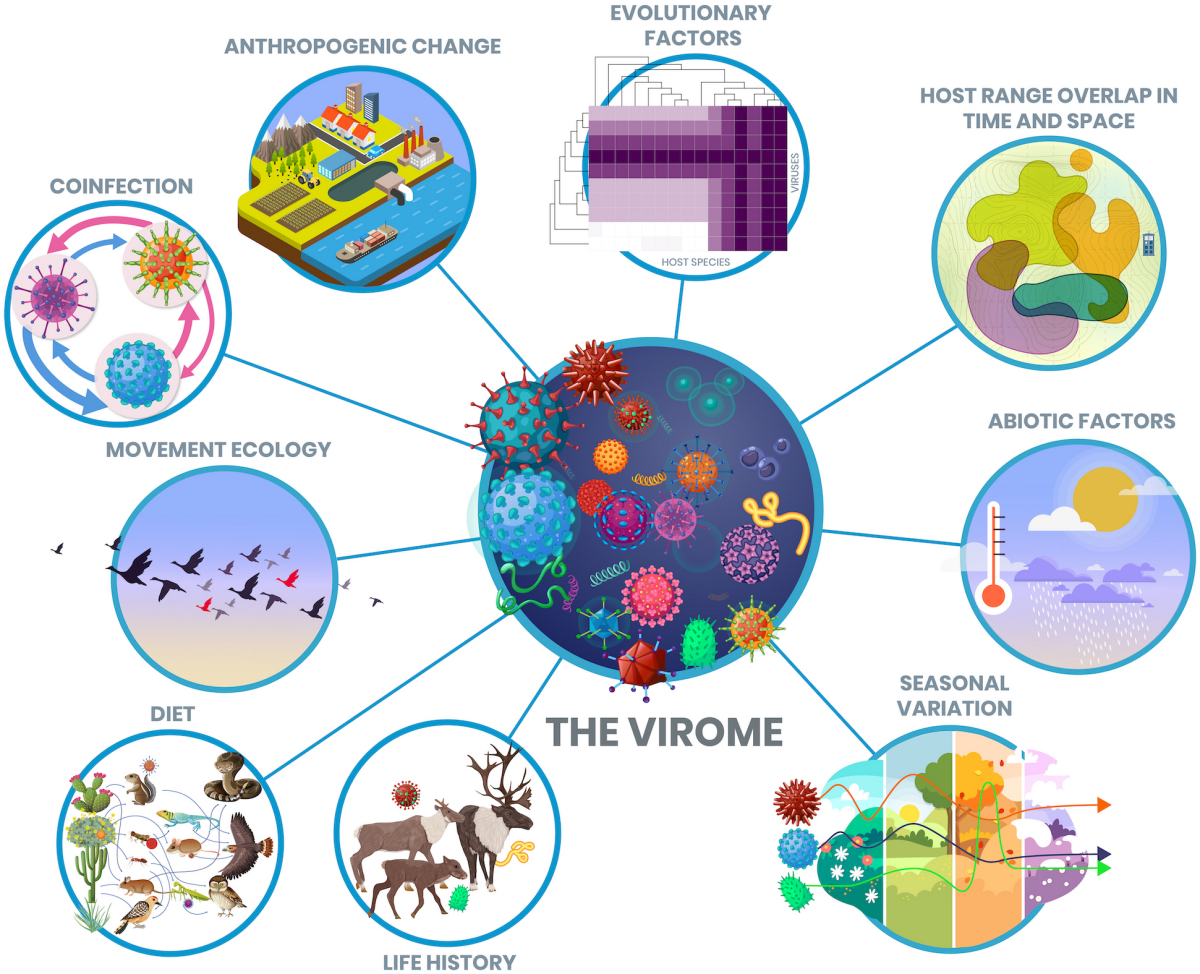
The ecological and evolutionary drivers of viromes. Viromes can be considered at multiple levels: individual organisms, populations, species or whole ecosystems. Factors influencing the virome may interact. For example, seasonal changes in host range may coincide with seasonal peaks in infection burden, with coinfection interactions shifting components of the virome.

Host ecological traits have a major impact on the composition and diversity of viromes, operating primarily through influencing the likelihood of exposure at multiple scales and interacting with each other and with evolutionary factors (figure 2). First, spatial and temporal differences in a host’s distribution affect the likelihood of exposure and virus sharing. Indeed, some studies have revealed a positive relationship between host geographic range overlap and the likelihood of viral sharing, cross species transmission and viral richness [13,17,49,64]. Second, within communities of sympatric organisms, biotic factors limit exposure between host individuals through food webs or trophic networks, dietary preferences, age structures and predator–prey networks. At both scales, anthropogenic driven climate and land-use change will alter host dynamics, with knock-on effects on virome composition and diversity. Within-host ecological interactions can also modulate the likelihood of virus acquisition [65], for example, through coinfection with other viruses or non-viral pathogens or through interactions with the resident microbiota. These interactions can alter infection outcomes and onward spread, and hence larger population level virus diversity, prevalence or abundance [29].

### (a) Abiotic associations with virome diversity and abundance

Key abiotic factors such as temperature, humidity and rainfall, all shape the prevalence of individual viruses by modulating host population behaviour or viral transmission/environmental persistence [66]. We might therefore expect that virus prevalence and diversity will follow similar trends to those seen in other microbes, exhibiting broad-scale elevational/depth and latitudinal gradients, with these abiotic factors driving changes in virome diversity and composition.

Ocean temperature modulates the abundance and composition of both marine bacteriophage communities [67] and the viral communities of fish [61]. In terrestrial organisms, increases in elevation are associated with a decline in viral richness in vampire bats, with colonies at lower elevations in the Amazon rainforest having higher viral richness and distinct community composition [68]. Given that host species richness generally increases towards the equator via the latitudinal diversity gradient, latitude (as well as longitude) has been identified as a modulator of virus communities, acting as a proxy for both the biotic and abiotic variables described above. For example, marine virus diversity showed higher diversity at lower latitudes, with decreasing virus diversity moving poleward, mirroring that of most aquatic and terrestrial host diversity patterns [69]. In addition, longitude is a significant factor in explaining virus diversity in bats [68], while both latitude and longitude had a very strong impact on the human gut virome even when accounting for ethnicity and other demographic factors [70]. However, in contrast to these clear latitudinal and longitudinal trends, the viruses infecting fish species and individuals in Antarctica are just as diverse and abundant as those from warmer marine environments [71], despite the host diversity gradient. It may therefore be that our relative lack of knowledge on virus diversity in many groups obscures caveats to the assumption that virome diversity increases with host diversity. For example, phylogenetic rarity (the phylogenetic distance between species in a community) may be more important in determining virome diversity, and temperate areas may facilitate larger aggregations of species, increasing contact rates and the transmission of viruses [72].

#### (i) Seasonal variation in viromes

If temperature, humidity and rainfall can drive species viral diversity and composition, then viromes will vary seasonally, particularly in temperate regions. Indeed, seasonal trends in virus prevalence have been observed with individual viruses [73], particularly with respiratory viruses, with the highest prevalence in autumn months [74]. From the few virome studies which have addressed seasonality, viral prevalence, evenness and richness also display seasonal trends [75]. Additionally, in surveys of wastewater, viral alpha and beta diversity varies significantly by season [76]. However, in a study of seasonality in the *Picornaviridae* component of rodent viromes, evenness peaks in spring/summer, pre-dating peaks in virus prevalence seen in autumn [77]. Indeed, the intrinsic link between seasonal trends and abiotic factors such as temperature, humidity and rainfall, daylight and biotic factors such as host immune response, physiology, movement or other behaviours, make the precise drivers of these trends both difficult to disentangle, and worthy of further detailed study (box 1). Even for the best-studied viruses of humans, such as influenza, we are only just beginning to unravel the complexity of seasonal trends [78].

### (b) Host biotic factors that shape virome composition

A number of potential biotic moderators of virus community diversity and structure have been identified. These include life-history traits, species migration histories and demography. This is an area of huge potential expansion into topics such as how social networks [79,80] and behaviour [81] impact virus transmission. Here, we will address four descriptive factors; the composition of the population by life-history traits (here host age and sex), host dietary preferences and a host species history of range movement/migration.

#### (i) Life-history traits

Host age is a key feature of virus susceptibility and host immune response, as, at least in vertebrates, young animals are more susceptible to viral infections, and show a consistently higher prevalence compared to adults [82–84]. At the level of whole viromes, host age is arguably the most studied demographic factor affecting virome diversity and composition, across a wide range of species, from humans [85] to echinoderms [86]. In the future, we need an increased understanding of the impact of host age structure on virome diversity and composition in invertebrates, where antibodies do not mediate susceptibility, and therefore could show vastly different trends.

In contrast to host age, host sex has not yet clearly been associated with whole virome composition [68,75,87]. From studies in some individual pathogens, it might be predicted that males will show higher prevalences of viruses due to behaviour and physiology, with knock-on effects on whole virome composition, perhaps decreasing alpha diversity. However, studies have not all shown a clear trend in this direction, perhaps reflecting the varied impact of host sex on individual viruses [88], lack of behavioural or immune differences between sexes in some systems [82], the taxonomic groups considered or a result of study design.

#### (ii) Diet

The impact of ecosystem food web structure on species virome diversity and composition is as yet unknown, despite viruses playing an integral part in food webs, the recycling of organic matter and transfer of energy across trophic levels [83]. At an individual level, studies of human gut viromes have provided some limited evidence that dietary variation can impact gut virome community structure [89], as it does for bacterial microbiomes [8]. However, at a broader species level, we do not know if certain types of diet, or a more phylogenetically diverse diet, drives higher virome diversity. Dietary preferences and increased dietary phylogenetic breadth could increase the opportunity for viral host shifts, and a more diverse virome. However, when predator–prey or herbivore–plant pairings are phylogenetically distant, current data from animal viromes suggests that viruses are not often shared during these interactions [90], and that host phylogeny plays a larger role in virus sharing [28]. In the future, the study of whole food webs, and multi-species, phylogenetically controlled comparisons, will enable the effect of species diet on virome diversity and composition to be better quantified. However, in studies of wild populations it is extremely important to distinguish between the transient gut virome, which are likely to be actually infecting dietary or prey species, and ‘resident’ viruses that cause sustained infections and go on to persist in their new host (box 1).

#### (iii) Movement ecology and virome composition

Species migration and dispersal, as well as their history of invasion or introduction, are likely to have significant impacts on current virome composition. The idea that an individual’s movement ecology and demographic history influence the prevalence and diversity of parasites is not a novel one [91]. To date, however, such factors have rarely been considered in studies of virus ecology.

There is a pressing need to understand the role of species’ histories of introduction and dispersal in shaping the current virome, given how rapidly distributions are shifting in tandem with climate change, and the frequency of introductions via global trade and travel [92]. For example, increasing ocean temperatures are likely to drastically shift marine species’ distributions [93]. As these ranges shift poleward in response to changing climates, species will be pushed into contact with novel viruses [94]. They will also expose native and naive host species to novel viruses, perhaps with devastating consequences. Species invasions may also change species–virus relationships, and the diversity of the whole host ecosystem—with potential knock-on effects such as the ‘dilution effect’ [95]. The outcome of these species–virus interactions will also be influenced by the phylogenetic relationships between hosts, including their evolutionary rarity (i.e. how phylogenetically distant a host is from the rest of the host community). For example, introduced hosts that are phylogenetically isolated from other members of the host community have lower disease pressure [96]. However, we lack a comprehensive understanding of whether dispersing individuals act as sources or sinks for viral infection, and how these patterns vary with host taxonomy. For instance, do recent arrivals in an ecosystem exhibit reduced virus diversity or abundance, or do they tend to act as sources of novel viruses?

Species movement also affects virome composition through the life-history strategy of migration. For example, host migration may lead to escape from pathogens (with small founding populations less likely to carry acute infections), to infected individuals being removed from populations and to recovery from infection or spatially isolated infected and uninfected individuals [97]. Studies of viruses such as avian influenza and West Nile virus have shown that migration might have increased the spread of disease in general by increasing contacts and, thus, virus exposures [74]. Future work should investigate how migratory strategies shape variation in both virome composition and risk of transmission at the individual level.

### (c) Anthropogenic factors that influence virome community structure

Human driven changes to the natural environment, such as climate change, urbanization, habitat disturbance and altered nutrient cycling, have a profound impact on host biodiversity, and alter the ecology of systems governed by the abiotic and biotic factors described above. However, we poorly understand the broader consequences of such changes to virome community diversity.

Studies in humans suggest that urbanization can have a profound impact on the diversity and composition of viromes [98,99], with important differences observed between the viromes of urban and rural-living humans [70,100]. An important caveat is that, to date, studies involving humans have largely focused on the gut virome (i.e. bacteriophages) rather than viruses that infect human cells, so what we are observing could be driven by differences in the bacterial microbiome.

In wild populations, anthropogenic factors have a profound impact on the distribution and home ranges of many host species, with the potential to facilitate the cross-species transmission of viral pathogens, affecting wildlife conservation, agriculture and human health [101]. While few studies have assessed the impact of host biodiversity changes on the virome, our limited evidence suggests some pristine/undisturbed habitats have increased viral diversity, likely related to an increase in host species diversity in some systems [102].

### (d) Coinfection as a modulator of species viromes

Coinfections, in which a host is simultaneously infected with multiple viruses, parasites or symbiotic microbiota, are common in nature and may alter the outcomes of individual infections [25]. Within coinfected hosts, viruses can interact directly—such as in the transactivation of one virus’s gene expression by the proteins of another [103]. Similarly, viruses can interact indirectly (with other viruses, microbes or parasites) through modulation of host components such as immune activity or resource availability [104]. These interactions can create synergistic, exploitative or competitive relationships between pathogens.

There is not yet a clear link between coinfection interactions, coinfection prevalence and virome composition. However, the consequences of interactions between pathogens in individual hosts can affect the prevalence of viruses across host populations. For example, negative interactions between influenza A virus and Rhinovirus in humans can lead to fluctuating and asynchronous seasonal prevalences of each virus [105], and has been suggested to have delayed the introduction of the 2009 H1N1 influenza virus pandemic to Europe [106,107] (after which H1N1 is thought to have disrupted the epidemic transmission of another respiratory virus [108]).

Compared to single infections, coinfections can alter the relative fitness of different viruses and virus genotypes [109,110], and may play a role in generating and maintaining virus diversity. For example, in nucleopolyhedrovirus coinfections of pine beauty moths (*Panolis flammea*), the relative fitness of virus genotypes during single infection does not correspond to fitness during coinfection, and are further influenced by the ecological context of the infected host [111]. At a population level, coinfection-induced changes in the rank order of virus fitness are expected to fluctuate with coinfection prevalence [112]. Additionally, the outcome of coinfections is likely to be heavily influenced by the sequential timing of infections [113], with within-host viral diversity sometimes dependant on the order in which viral infections occur. In this way, coinfection may be an important—yet relatively understudied—mechanism for the maintenance of virus diversity and the shaping of host viromes [114].

To date, virome studies have often had limited opportunity to study coinfections due to pooling of samples from multiple individuals. However, reduced sequencing costs are now making single individual sequencing libraries increasingly viable (box 1), although an enormous amount of data will be generated. As such, more studies will be able to examine the role coinfections and interactions between viruses play in driving virome composition within hosts, and how this determines population level dynamics. Metagenomic approaches also allow for the inference of co-occurrence and interaction networks between viruses and any organisms sequenced alongside them, allowing viromes to be linked to the wider microbial community within a host. For example, in a study of *Ixodes* ticks, positive associations were detected between multiple virus species, the causative agent of Lyme disease (*Borrelia burgdorferi*) and *Rickettsia* sp. [115]. In humans, the presence of *Pseudomonas* bacteria in lung tissue is both positively and negatively associated with multiple viruses, and the direction of this interaction can change depending on individual co-morbidities [116]. Integrating these networks with environmental data may ultimately allow for a greater understanding of how microbial and ecological contexts combine to influence virome composition and dynamics [117].

## 4. Perspectives

Although we have attempted to synthesize the current evidence on what drives the diversity and composition of species viromes, the majority of data still come from single-host, single-virus studies. Such studies may not generalize to whole virus communities, and could be focused on viral ‘oddities’ such as extremely virulent viruses, that are unlikely to represent the majority of the virome. With ever decreasing costs of RNA sequencing, hypothesis-driven and structured sampling of viromes from multiple host individuals, populations and species in a community, is becoming more affordable. As such, collecting high-quality data (box 1) to improve our understanding of the key ecological and evolutionary drivers of the virome is increasingly within reach.

Despite the unique challenges that virome studies bring, there are many exciting areas for expansion in this field, and many outstanding questions about the basic relationships driving the distribution of viruses across host species. For example, do areas with a greater diversity of host species generate higher virome diversity, or is this dependant on the phylogenetic composition of the host community? Are species with more diverse viromes more likely to acquire more viruses, and are generalist viruses more likely to infect new species than specialist ones [18]? At a population level, what is the relationship between population size and virome diversity [68]? This is particularly interesting to consider in the tropics, as numerous studies have shown the role temperature or UV play in virus transmission by reducing environmental persistence. Another unexplored aspect of the drivers of virome composition are social networks, and how associations within social networks drive virus transmission [80]. In the future, can we determine the mechanistic basis of the host–virus associations, in particular, the phylogeny-related variation? Can we use trait, gene or motif-based models/phylogenies of viruses to test the predictive power of these features in driving the distribution of viruses? Perhaps we can also move towards a more holistic, whole-microbial community approach to these studies, with exciting opportunities to study covariation among viruses, bacteria and fungi across a broad host phylogeny [37].

These questions are particularly timely due to ongoing global and climate change. Will increasing urbanization and global movement drive an increase in the virome diversity of the urban populations of wildlife, or a decrease in virome diversity due to lower host diversity? With global changes in non-urban areas, such as conversion to monoculture, what are their impacts on virome diversity downstream? Or, as in the case of habitat fragmentation [98], will the break-up of diverse ecosystems result in increased prevalences for the most abundant viruses and a corresponding reduction in virome evenness?

By understanding the evolutionary and ecological drivers of the virosphere, particularly the proliferation of zoonotic pathogens through communities and landscapes, we can also provide data that will help mitigate these risk factors. For example, methods of reducing the prevalence of harmful viruses, such as reducing the prevalence of vector-borne viruses through dilution effects (selectively increasing livestock densities), have been proposed [118]. However, their effectiveness will depend on the degree to which virus prevalence is driven by specific host densities, and how this changes with local spatial and temporal variation in abiotic factors. In addition, obtaining sufficient data on drivers of virus abundance to forecast or predict outbreaks will be challenging. Perhaps a more achievable short-term goal is to develop clear rules of thumb to build qualitative frameworks for understanding the ecology and evolution of the virome. With a greater understanding of the drivers of virus dynamics, we can aim to control viruses with impacts on human, agricultural and wildlife health, and also understand the role viruses play as components of whole ecosystems.

## Data Availability

This article has no additional data.
